# Fatal air embolism as complication of percutaneous dilatational tracheostomy on venovenous extracorporeal membrane oxygenation, two case reports

**DOI:** 10.1186/s13019-016-0489-9

**Published:** 2016-07-11

**Authors:** Achim Lother, Tobias Wengenmayer, Christoph Benk, Christoph Bode, Dawid L. Staudacher

**Affiliations:** Department of Cardiology and Angiology I, Heart Center Freiburg University, Hugstetterstrasse 55, 79106 Freiburg, Germany; Department of Cardiovascular Surgery, Heart Center Freiburg University, Freiburg, Germany

**Keywords:** ECMO, Extracorporeal membrane oxygenation, Complication, Air embolization, Percutaneous dilatational tracheostomy, Case report

## Abstract

**Background:**

Tracheostomy is recommended in case of prolonged mechanical ventilation. Therefore, most patients with an indication for venovenous extracorporeal membrane oxygenation (ECMO) will also have an indication for tracheostomy.

**Case presentation:**

We report 2 cases of fatal air embolism into the ECMO system as complication of percutaneous dilatational tracheostomy. Both patients had an AVALON ELITE® bi-caval cannula implanted draining blood from the vena cava superior and inferior.

**Conclusion:**

Since there is limited safety data on this specific group of patients, a routine early dilatational tracheostomy might be associated with a significant risk.

## Background

Tracheostomy is recommended in critical ill patients when prolonged mechanical ventilation is presumed [1]. Optimal timing of tracheostomy is still under debate [1, 2]. However, a recently published meta-analysis suggests that performing tracheostomy within 7 days after intubation may reduce intensive care unit stay [2]. Most studies comparing early to late tracheostomy defined late tracheostomy as performed in week two after intubation [1, 2]. Venovenous extracorporeal membrane oxygenation (vv-ECMO) is a treatment option for severe adult respiratory failure [3]. The average time on mechanical ventilation of vv-ECMO patients is 23 days [4]. Therefore most vv-ECMO patients will have an indication for tracheostomy.

We have to report 2 cases of fatal air embolism into the vv-ECMO in patients undergoing percutaneous dilatational tracheostomy while being on full ECMO support.

## Case 1

A 65 year old male without significant comorbidities presented at a non-ECMO hospital with H1N1 pneumonia. As a severe ARDS developed (Horovitz index 58) a vv-ECMO was implanted via the right jugular vein using a 31 Fr AVALON ELITE® bi-caval catheter and the patient was transferred to our intensive care unit. Six days after ECMO initiation, the patient was still ECMO dependent (blood flow 3.9 l/min, gas flow of 5.0 l/min) while on invasive mechanical ventilation (FiO2 45 %, PEEP 15 mbar). We therefore presumed a prolonged weaning and aimed for a percutaneous dilatational tracheostomy using the ULTRAperc system (Portex®, Smith medical, England) with bronchoscopic guidance. After puncture of the trachea and immediately after the first dilation step a significant air embolism into the ECMO system was observed. The ECMO system was halted but air could not be removed. Invasive mechanical ventilation failed to achieve sufficient oxygenation and hypoxic cardiac arrest occurred. After 20 min of cardiopulmonary resuscitation the patient could be stabilized after implantation of a new ECMO system. Even though primary resuscitation was successful the patient never recovered from the event and died on ECMO at day 27.

## Case 2

A 57-year old male was admitted to hospital for acute interstitial lung disease. He developed progressive combined hypoxic (Horovitz index 54) and hypercapnic respiratory failure. Venovenous ECMO therapy was established using a 27 Fr bi-caval catheter (AVALON ELITE®, Maquet, Germany). A combined immunosuppressive therapy with cyclophosphamide and prednisolone was initiated. After 10 days of mechanical ventilation (fiO2 45 %, PEEP 9) and 4 days on vv-ECMO (blood flow 4.5 l/min, gas flow 7.0 l/min) we performed dilatational tracheostomy using the 8.0 Fr Ciaglia Blue Rhino® percutaneous introducer set (Cook medicals, USA). Stab incision and sequential dilation caused minor venous bleeding. The dilator was reintroduced and external compression applied. Within seconds air embolism into the ECMO system occurred. Rapidly, the patient developed hypoxemia leading to cardiac arrest. Despite immediate resuscitation and implantation of a new ECMO system via the femoral veins return of spontaneous circulation could not be achieved.

## Discussion

Percutaneous dilatational tracheostomy is a frequent intervention in critical ill patients on the intensive care unit. In large case series, mortality rates range from 0.17 % [5] to 0.60 % [6]. Most of these fatal complications however were not directly associated with tracheostomy. Case series of fatal complications directly related to percutaneous dilatational tracheostomy [7] report damage of large arteries, uncontrollable bleeding or airway loss. A significant air embolization after surgical tracheostomy was reported earlier [8] but did not reach significant levels in a meta-analysis including over 113.000 procedures [6].

In a recent review, 30 out of 168 patients underwent tracheotomy while on ECMO therapy [4] with a significant variation between different centers (ranging from 4 to 46 % of all ECMO patients). No data is available concerning ECMO cannulation (bi-femoral, femo-jugular or bi-jugular) [4]. A case of a fatal complication directly related to tracheostomy in ECMO patients has not been reported so far. Bleeding, the most frequent complication of tracheostomy [4, 6, 7], however might be even pronounced in ECMO patients due to activation and consumption of coagualiton enzymes, platelet depletion and anticoagulant medication [9].

**Fig. 1:**
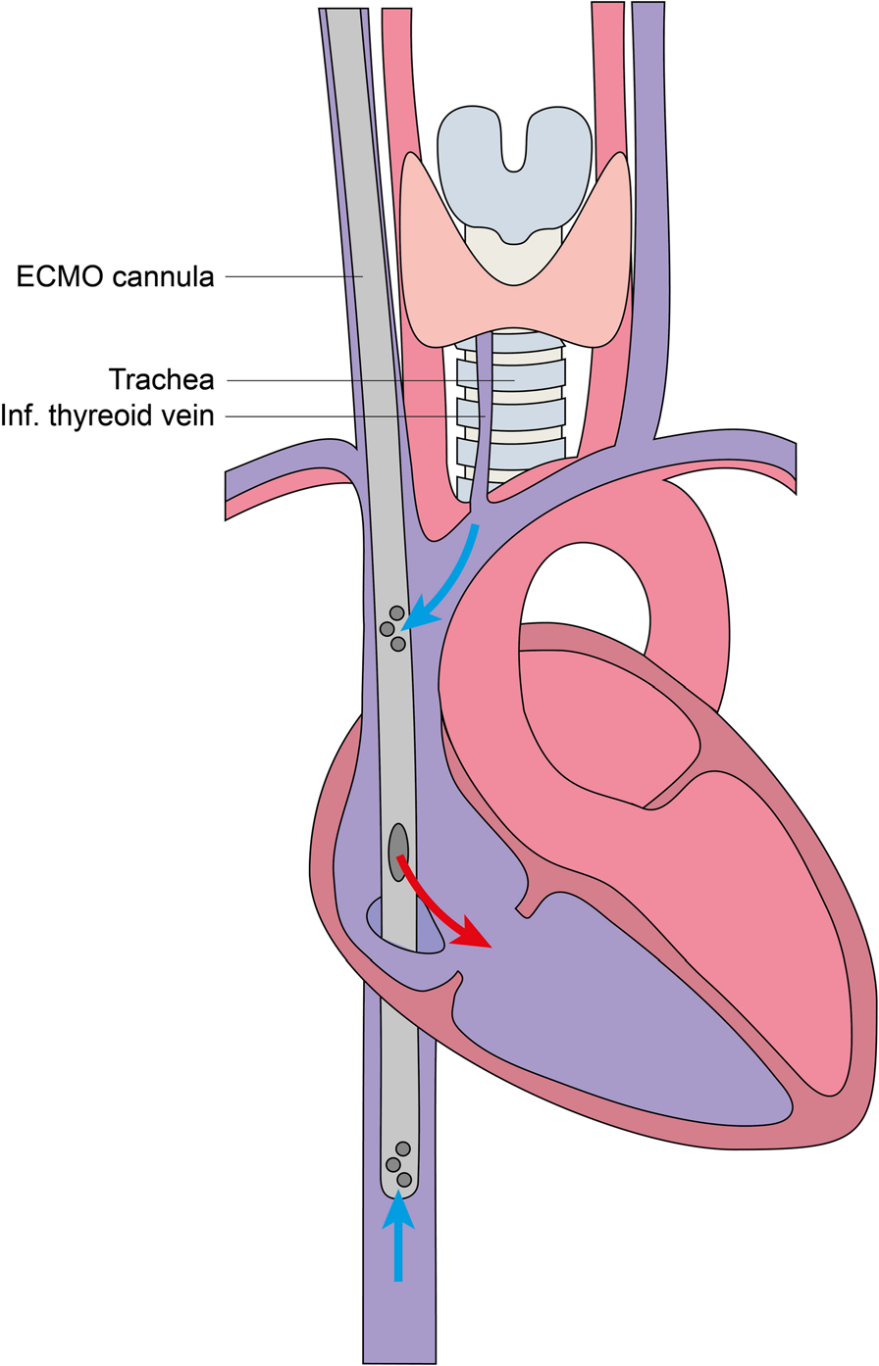
Air embolization into the ECMO system during tracheostomy. The AVALON ELITE bi-caval cannula drains venous blood from the superior as well as the inferior vena cava. Since the inferior thyroid vein directly drains into the vena cava superior, a venous damage during tracheostomy might result in severe air embolization into the ECMO

In the 2 patients presented here, the air embolization into the ECMO was detected seconds after dilation with a percutaneous tracheostomy dilator. Both patients were on full ECMO support via an AVALON ELITE® (Maquet, Germany) bi-caval dual lumen cannula draining blood directly from the superior vena cava (Fig. 1). Especially with higher ECMO blood flow, this might result in significant negative pressure in the draining vessels. We therefore hypothesize that suction of air through the inferior thyroidal vein draining blood directly to the vena cava superior might be responsible for fatal air embolism in both patients.

During the time period between 2013 to 2015, we performed a total of 30 percutaneous dilatational tracheostomy in patients on ECMO therapy, of which 24 had an Avalon elite cannula. As a routine measure, an ultrasound study is performed in all our patients in order to rule out large vessels at the puncture site before proceeding to dilatational tracheostomy. Since bleeding incidence is comparable in surgical versus dilatational tracheostomy [1, 10] it appears unlikely that surgical tracheostomy could have prevented these complications.

In response to the incidents described above we implemented the following points before performing a tracheostomy in ECMO patients on AVALON ELITE® cannulas.

- In case of severe hypoxia without ECMO (using 100 % FiO_2_ at the respirator) delay tracheostomy.
- During tracheostomy reduce ECMO blood flow (and thus negative suction pressure) as far as possible.
- Perform tracheostomy in a head down position.
- Cover puncture site at all times with wet compresses.

## Conclusion

There is limited data on safety of percutaneous dilatational tracheostomy in ECMO patients. Especially in patients on full ECMO support using the AVALON ELITE® bi-caval dual lumen catheter dilatational tracheostomy might be associated with a significant risk.

## Abbreviations

ECMO, extracorporeal membrane oxygenation
